# Real-time swelling-collapse kinetics of nanogels driven by XFEL pulses

**DOI:** 10.1126/sciadv.adm7876

**Published:** 2024-04-19

**Authors:** Francesco Dallari, Irina Lokteva, Johannes Möller, Wojciech Roseker, Claudia Goy, Fabian Westermeier, Ulrike Boesenberg, Jörg Hallmann, Angel Rodriguez-Fernandez, Markus Scholz, Roman Shayduk, Anders Madsen, Gerhard Grübel, Felix Lehmkühler

**Affiliations:** ^1^ Deutsches Elektronen-Synchrotron DESY, Notkestr. 85, 22607 Hamburg, Germany.; ^2^ Department of Physics and Astronmy, University of Padua, Via Marzolo 8, 35131 Padova, Italy.; ^3^ The Hamburg Centre for Ultrafast Imaging, Luruper Chaussee 149, 22761 Hamburg, Germany.; ^4^ European X-Ray Free-Electron Laser Facility, Holzkoppel 4, 22869 Schenefeld, Germany.

## Abstract

Stimuli-responsive polymers are an important class of materials with many applications in nanotechnology and drug delivery. The most prominent one is poly-*N*-isopropylacrylamide (PNIPAm). The characterization of the kinetics of its change after a temperature jump is still a lively research topic, especially at nanometer-length scales where it is not possible to rely on conventional microscopic techniques. Here, we measured in real time the collapse of a PNIPAm shell on silica nanoparticles with megahertz x-ray photon correlation spectroscopy at the European XFEL. We characterize the changes of the particles diffusion constant as a function of time and consequently local temperature on sub-microsecond timescales. We developed a phenomenological model to describe the observed data and extract the characteristic times associated to the swelling and collapse processes. Different from previous studies tracking the turbidity of PNIPAm dispersions and using laser heating, we find collapse times below microsecond timescales and two to three orders of magnitude slower swelling times.

## Teaser

The time and temperature evolution of PNIPAm nanogel was studied at sub-μs timescales with XPCS performed at the European XFEL.

## INTRODUCTION

Stimuli-responsive hydrogels are a class of materials that undergo a volume phase transition as a response to variations in their local environment, such as changes in pH, pressure, or temperature, enabling a wide range of applications from nanorobotics to drug delivery or adaptive optics (*1*–*3*). Collapse-inducing stimuli weaken the polymer-solvent interactions and strengthen the polymer-polymer ones, leading to a partial expulsion of the solvent from the gel. Many micro- and nano-gels are based on poly-*N*-isopropylacrylamide (PNIPAm) in water. While the equilibrium response of such systems has been intensively investigated (*1*, *4*–*6*), it is only in recent years that the kinetics of the collapse mechanisms have been studied in time-resolved experiments providing previously unknown information on the details of these processes (*7*, *8*). For PNIPAm, the collapse and swelling can be triggered by different stimuli: It can be induced by temperature (*7*, *9*–*13*), cononsolvency effects (*8*, *14*), and pressure (*15*–*18*). The knowledge of the timescales for this volume phase transition is very important for potential applications, especially for microgels, i.e., systems approaching the micrometer- or nanometer-length scales. For hydrogel particles with radii in the millimeter to micrometer range, the swelling-collapse kinetics is reasonably well described by the Tanaka model (*19*, *20*), which predicts a characteristic time τ_c_ proportional to the square of the radius of a fully swollen particle. This sort of experiment is usually performed by inducing a temperature jump of the system and following the time evolution of a certain observable, e.g., optical density (*13*) or absorption spectrum (*7*, *12*). In particular, if the energy is delivered first to the solvent, as, e.g., in the capacitor-discharges of (*13*), then the microgel undergoes a two-step collapse which is attributed to the formation of an outer layer of collapsed polymer which slows down the escape of the water molecules trapped inside. Conversely, if the particles are heated directly, e.g., using Au core–PNIPAm–shell systems as in (*12*) and a pump laser tuned to the plasmonic resonance of the core particle, then a single-step process is observed. The latter process shows markedly faster collapsing and swelling times (*7*).

Here, we perform a direct measurement of the hydrodynamic radius of silica-PNIPAm core-shell particles dispersed in water. We apply megahertz x-ray photon correlation spectroscopy (XPCS) using the x-ray pulse trains of the European X-Ray Free-Electron Laser Facility (EuXFEL) to measure the diffusion properties of the dispersion. Nanoparticles dispersed in liquids undergo Brownian motion with a diffusion constant (*D*) determined by the Stokes-Einstein relation via

$$D=\frac{k_{B}T}{6\pi R\eta \left(T\right)}$$ (1)

where *k*_B_ is Boltzmann’s constant, *T* is the temperature, η(*T*) is the temperature-dependent viscosity, and *R* is the particle’s hydrodynamic radius, which for PNIPAm is also a function of temperature. Measuring the diffusion constant is therefore an effective way to track the swelling or collapse of the polymers, as commonly done in dynamic light scattering (DLS) experiments (*1*, *4*, *5*). XPCS is the x-ray analog to DLS (*21*). Because of the shorter wavelengths, XPCS enables studies at much larger wave vector transfers covering simultaneously length scales spanning from micrometers to nanometers in small-angle x-ray scattering (SAXS) configuration. In particular, it has been recently applied to study the phase behavior of PNIPAm systems (*22*–*24*). The development of x-ray free electron lasers (XFELs) with repetition rates in the megahertz regime enabled the study of sub-microsecond processes (*25*–*27*). This is, for instance, the natural diffusion timescale of nanometer-sized particles dispersed in water.

Furthermore, XPCS at the EuXFEL allows following the time evolution of driven and out-of-equilibrium systems over time and length scales that cannot be reached with other more conventional experiments. In this scheme, the ultrashort x-ray pulses act both as pumps heating the sample and as probes of the dynamics with sub-microsecond time resolution. Here, we present results from x-ray heating induced collapse and swelling of the PNIPAm shell at different cross-linker concentrations and different shell thicknesses. An increasing number of research focus on real-time dynamics of biological systems at free electron laser (FEL) facilities (*27*, *28*). FELs make possible to have a direct and real-time view on phenomena such as protein folding kinetics or phase separation, but, at the same time, pose important challenges in the form of radiation-induced effects and sample damage. Therefore, it is crucial to understand the response of soft and biological matter under such intense and energetic photons on microsecond timescales. Depending on the softness of the particles, the x-ray pulse fluence and repetion rate, we observe different dynamical domains corresponding to swollen particles, collapsed particles, as well as silica cores with radiation-induced loss of the PNIPAm shell. We find two timescales governing the dynamics of PNIPAm. A fast collapse time in the range of 100 ns and a slow swelling time of τ_s_ > 100 μs. Compared to previous studies focusing on indirect quantities such as optical density, we obtain a more direct measurement of the (de)swelling process by accessing their diffusion properties on intrinsic microscopic length scales.

## RESULTS

**Fig. 1.**
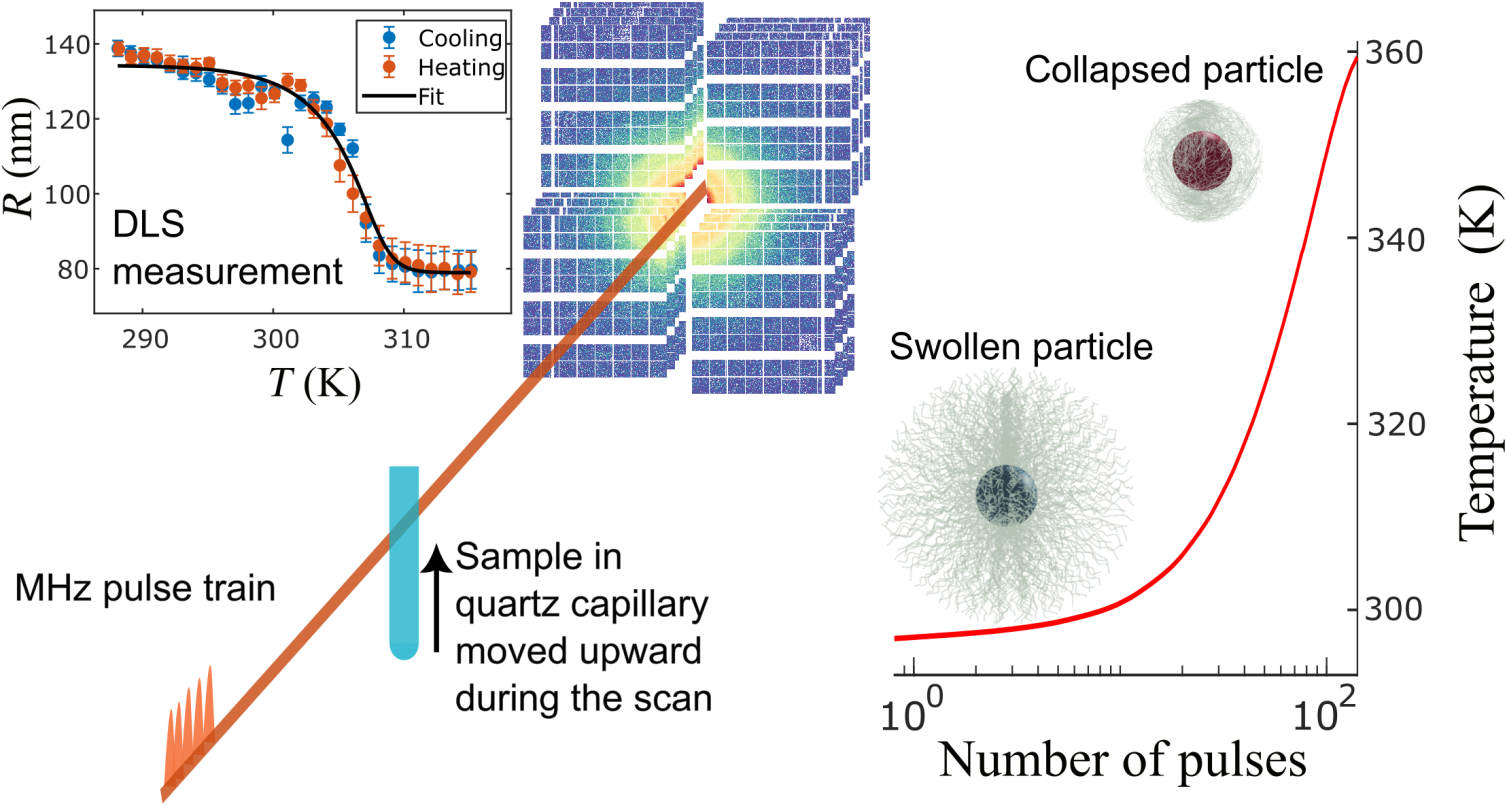
Conceptual scheme of the experiment. Center: The train of x-ray pulses reaches the sample and heats it up in a controlled manner (red line in the right plot). If the silica nanoparticles are covered by PNIPAm, then the series of pulses will trigger the transition from the swollen hydrophilic to the collapsed hydrophobic state. In the top left plot, a typical DLS characterization of one of our core-shell systems is displayed, specifically a sample at high cross-linker concentration and thin shell thickness of ∼35 nm in the collapsed state.

The samples were probed by trains of 9 keV x-ray pulses with repetition rates of up to 2.24 MHz; see Materials and Methods for details. Because of the high repetition rate of intense x-ray pulses, the samples experience sequential heating. A fast heating takes place as response to the first pulse, followed by cooling before the next pulse hits the sample volume. Over the pulse train, a successive heating can be observed for certain dose-fluence regimes (*25*, *26*, *29*). Thus, the PNIPAm shell’s collapse is expected to be triggered by this heating, as schematically represented in Fig. 1. The systems studied here are composed of silica core–PNIPAm–shell nanoparticles synthesized with two different cross-linker concentrations, 4 and 8 wt % of methylenbisacrylamide (BIS) and two different shell thicknesses (~125 nm for the thinner and ~160 nm for the thicker shell); see Materials and Methods and Supplementary Materials for more details. The systems have been characterized by DLS to track the variation of the particles’ radii with temperature; see the inset in Fig. 1. These DLS measurements showed a lower critical solution temperature (*T*_lcst_) of ∼312 K for all samples. Because the difference in electron density between the polymer and water is negligible compared to the one between water and silica, the information of the scattered intensity *I*(*q*), where *q* is the exchanged wave vector, is dominated by the silica core in the accessible *q*-range (see the Supplementary Materials). Even after the complete collapse of the PNIPAm shell, it shows only small changes in a region with very low count rates, and consequently is affected by substantial systematic errors. Therefore, all the information on the kinetics of the microgel collapse is encoded in the changes in the dynamics of the particles.

### Effects of fluence and controlled heating

**Fig. 2.**
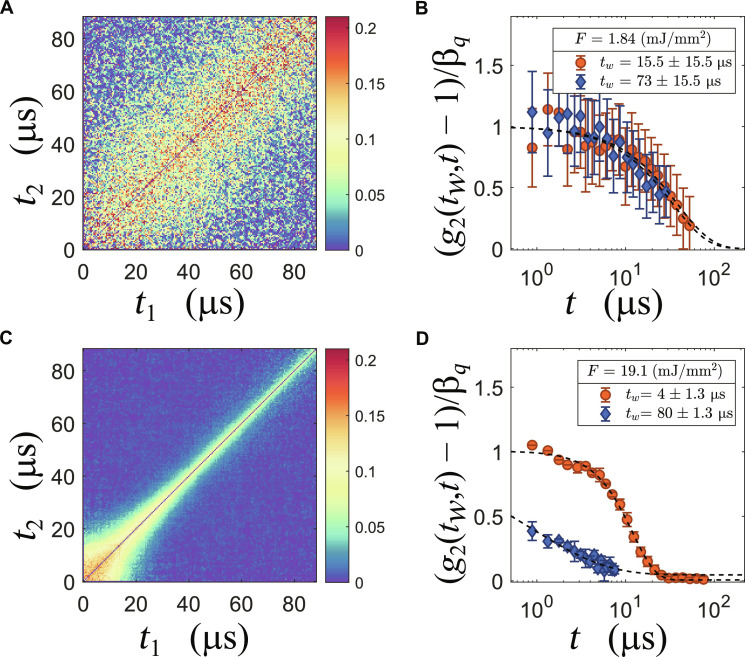
Examples of the observed dynamics at *q* = 0.087 nm^−1^ in two fluence regimes. (**A** and **C**) The TTCM for a low fluence (1.84 mJ/mm^2^) and a high fluence (19.12 mJ/mm^2^) measurement. (**B** and **D**) The corresponding time-resolved correlation functions *g*_2_(*t_w_*, *t*) at two *t_w_*: Orange circles show a *t_w_* at the beginning of the train, and blue diamonds show a *t_w_* at the end of the train. To fit the blue points in (D), both baseline and contrast were kept fixed at the values obtained from the fit at the beginning of the train.

The response of the nanoparticles is critically dependent on the x-ray intensity per pulse. Changes can be easily observed from the two-time correlation matrices (TTCMs) obtained by XPCS, which are a powerful tool to measure the nonequilibrium dynamics (*30*). Details of the XPCS approach used here are given in Materials and Methods. A broadening of the diagonal implies a slowing down of the dynamics in the scattering volume, as found, e.g., in aging glasses (*31*–*33*). Similarly, a narrowing implies a speeding up of the dynamical processes, as can be seen from Fig. 2C. Selecting sections of the TTCM, it is then possible to obtain a quantitative description of the nonequilibrium dynamics accessing information dependent on the time elapsed since the beginning of the measurement *t_w_*, which, in this context, means the time interval between the observed dynamical quantity and the first pulse that reached the sample. The most common way to characterize this time-dependent dynamics is to fit a Kohlrausch-Williams-Watts (KWW) function *g*_2_(*t*) − 1 = β*_q_* ⋅ exp [−(*t*/τ)^γ^] to the data, where τ is usually a function of *q* and eventually of *t_w_*. For diffusive systems γ ≈ 1 and τ(*q*) ∝ *q*^−2^. In systems such as the ones investigated here β*_q_* is a quantity that depends on the instrument parameters (*34*) that have been characterized elsewhere (*35*). In Fig. 2A, we report the TTCM for a sample with 8 wt % and thick shell taken at *q* = 0.087 nm^−1^ and fluence of *F* = 1.85 mJ/mm^2^ per pulse (here all fluence values are reported per pulse). As the width of the diagonal is constant at this fluence regime the dynamics of the particles remains stationary within a train. This is confirmed by the time-resolved intensity-intensity correlation functions *g*_2_(*t_w_*, *t*) starting at different time points *t_w_* within the pulse train; see Fig. 2B. The data shown here were obtained by averaging the first and last 30 μs of the TTCM. Despite the noisiness of the data points, a consequence of the low pulse intensity, it is possible to see how the sample relaxes following the same single exponential behavior at the beginning and at the end of the train. We conclude that, at least up to a certain fluence of about 2 mJ/mm^2^, the system remains at equilibrium and the change in temperature is too weak to be detected by the change in the dynamics at the probed length scales. Therefore, for measurements, in this fluence regime, the relaxation time only depends on *q*.

Increasing the fluence to 19.1 mJ/mm^2^, the TTCM shows a completely different behavior as shown in Fig. 2C. The dynamics sharply speeds up in less than 100 μs in a nonlinear fashion. In Fig. 2D, the corresponding *g*_2_(*t_w_*, *t*) functions obtained from slices averaged over 10 pulses (2.6 μs) at *t_w_* = 4 μs and *t_w_* = 80 μs are displayed. The relaxation in the first part of the train is described by a KWW function with a compressed exponential with value of γ = 1.65, indicating that the dynamics is better described by a ballistic motion than diffusion. Toward the end of the train, the apparent drop in contrast is due to a faster relaxation combined with a stretched KWW exponent (γ ≈ 0.5), indicating a wide distribution of relaxation times in the same scattering volume. Qualitatively, this situation is similar to what has been observed already in suspensions of pure silica nanoparticles and could be connected to heating of the particles and the surrounding water (*25*), where, for more intense fluences, the dynamics was markedly faster already after few pulses.

**Fig. 3.**
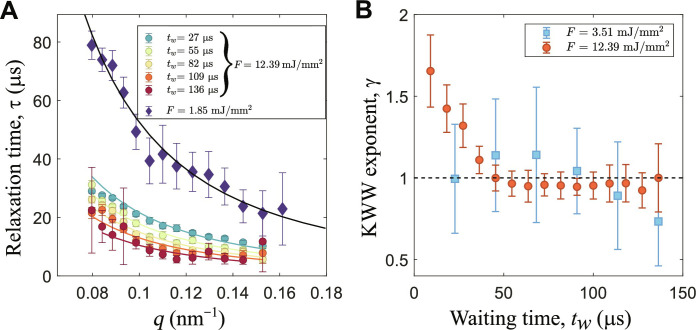
Relaxation times and KWW exponents for samples with thin shell and BIS 8 wt %. (**A**) Relaxation times as a function of *q* for two fluences. For *F* = 1.85 mJ/mm^2^, the average over the whole TTCM is performed. The black line is the fit of the Stokes-Einstein relation and returns a diffusion constant of 1.9 ± 0.1 nm^2^/μs. The colored data are the pulse-resolved average relaxation times for *F* = 12.2 mJ/mm^2^. (**B**) KWW exponent at different *t_w_* for two fluences of *F* = 12.2 mJ/mm^2^ and *F* = 3.51 mJ/mm^2^.

To extract more detailed information from the correlation functions, we analyzed the KWW shape parameter γ as a function of *q* and *t_w_* for different fluences. In Fig. 3A, the average relaxation time <τ(*q*)> = Γ_0_(1/γ)τ(*q*)/γ, where Γ_0_ is the gamma function, is reported for two runs at two representative fluences. The low fluence does not produce strong temperature changes within a train and, consequently, the relaxation times follow the Stokes-Einstein relation with a diffusion constant of 1.9 ± 0.1 nm^2^/μs, compatible with a swollen particle at 303 K. Conversely, the high fluence data show a decrease of the relaxation times for increasing *t_w_* (or number of pulses *n*_p_). In Fig. 3B, the shape parameters of the relaxation functions at different *t_w_* are reported for both fluences. While the data at lower fluences do not deviate from the exponential shape (i.e., γ = 1 expected for freely diffusing particles), we observe for larger fluences a γ > 1 for the initial pulses and a normal diffusive regime after few pulses, indicating a change of the relaxation from compressed to simple exponential and eventually to stretched, as discussed above. This suggests that the initial pulses induce a ballistic-like motion of the nanoparticles that quickly subsides in a diffusive behavior at later stages. The transition to a stretched exponent indicates the presence of a large distribution of relaxation times within the scattering volume. In a dilute system, as the one probed here, this means that each particle diffuses with a different diffusion constant. A similar behavior was observed for pure silica particles (*25*) and was attributed to the profile of the incident radiation and thus a heterogeneous temperature in the probed volume. However, sample damage is always present to a certain extent in XFEL experiments, even more in situations in which the same volume is exposed to sequences of pulses like in the present case. With our experience from past XFEL campaigns, we learned how to tackle beam induced effects (*25*, *26*). In addition, our knowledge on PNIPAm-based systems measured at several synchrotron and FEL light sources, including the SPring-8 Angstrom Compact free electron LAser (SACLA) (*36*), allows us to discern which effects are due to structural damages or to temperature effects. A discussion on the details of sample damage in organic and biological systems is outside the scope of this work, and we will focus our attention on temperature effects and damage thresholds. To this end, we will analyze the changes in diffusivity as a function of *t_w_*.

### Diffusion constant and effective temperature

**Fig. 4.**
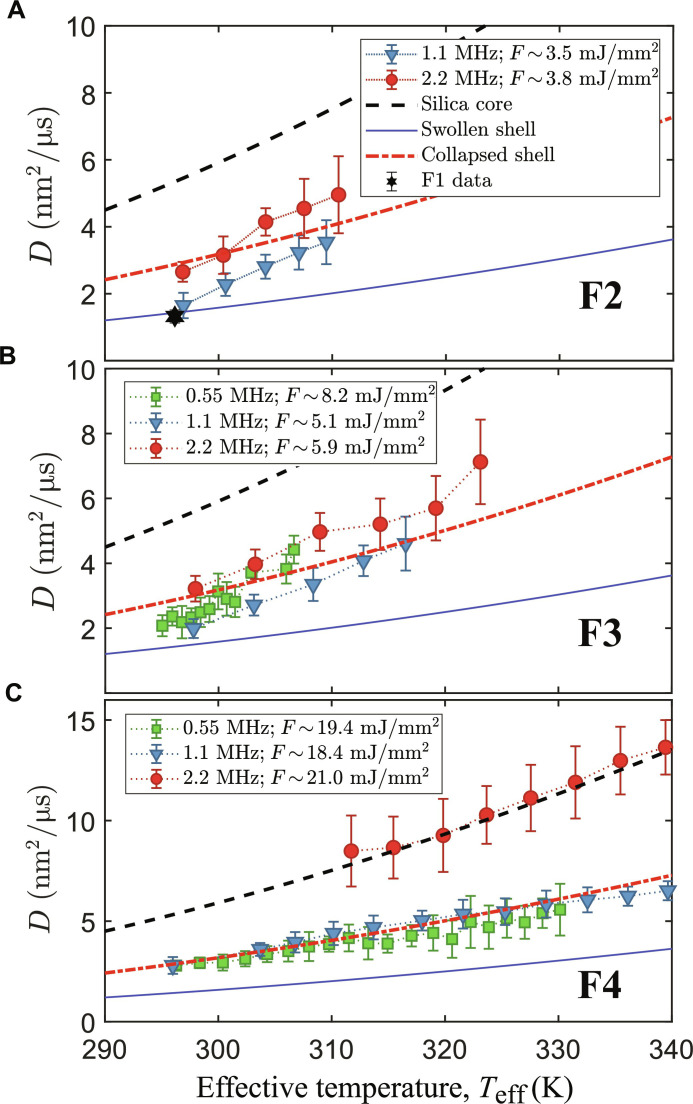
Diffusion constant for samples with thick shell and 8 wt % BIS as a function of* T*_eff_ for different repetition rates and different fluences. (**A**) Low fluence limit of regime F2, for comparison, the measured diffusion constant obtained from an F1 run is also reported (black star). Because of the limited signal-to-noise ratio, only data for 1.1 and 2.2 MHz are reported. (**B**) Intermediate fluences (regime F3). (**C**) High fluences (regime F4). The lines represent the limit values for the completely swollen (solid blue), fully collapsed (dash-dotted red), and pure silica core (dashed black) state.

The change of relaxation time is the combined effect of both the rising local temperature and the collapse of the PNIPAm shell. From the measurements of the relaxation time at different fluences, we can obtain the change of the diffusion constant *D* for different *t_w_* (or *n*_p_). As *D* is a function of temperature, both fluence and repetition rates contribute to the local “effective temperature” *T*_eff_ experienced by the particle (*25*, *26*). The key concepts at the basis of the “time-resolved” model are that the observed diffusion is affected by the changes in temperature and, consequently, viscosity, in the first few nanometers of water surrounding the particles produced by the heating of the particles themselves. The model, developed for aqueous dispersions of silica nanoparticles, can be used also in this situation under the assumption that the difference between the x-ray absorption coefficient of the hydrated PNIPAm shell and water is small and, therefore, neglegible. Using a known set of pure silica nanoparticles as a calibrant [see the Supplementary Materials and (*26*, *37*)], we evaluate the actual x-ray fluence delivered to the sample and thus the effective temperature. In the following, we distinguish four fluence regimes: regime F1 with *F* < 2 mJ/mm^2^, regime F2 with 2 mJ/mm^2^ ≤*F* < 5 mJ/mm^2^, regime F3 with 5 mJ/mm^2^ ≤*F* < 13 mJ/mm^2^, and regime F4 with *F* ≥ 13 mJ/mm^2^. As shown in Fig. 2, for F1, the diffusive dynamics do not vary over the pulse trains, suggesting no impact of beam-induced heating. Figure 4 shows *D* as a function of *T*_eff_ for one sample studied at three repetition rates and different fluences (regimes F2, F3, and F4). The experimental data are compared to the limit values, represented as lines in Fig. 4, that correspond to the diffusivity of completely swollen (blue solid line), fully collapsed (red dot-dashed line), and pure silica core (black dashed line) particles.

For F1, the average dynamics is stationary with only an increase of few degrees above room temperature and still below the *T*_lcst_. At moderate fluences (regime F2), reported in Fig. 4A, the diffusion constant deviates from the behavior of swollen particles. At 1.125-MHz repetiton rate, a transition from swollen to collapsed particles can be observed over the pulse train. In contrast, the diffusivities at 2.24 MHz are already close to the collapsed line at small *T*_eff_. This indicates that the particles have a smaller hydrodynamic radius, e.g., they have lost part of the PNIPAm shell. For regime F3, see Fig. 4B; again, the diffusivities at 2.24 MHz are close to, or above, the line of fully collapsed particles, while the 1.125-MHz data change from swollen to collapsed. The diffusivities measured at 0.55 MHz reach the line of collapsed particles at lower *T*_eff_ than that at 1.125 MHz. Figure 4C shows data for fluence regime F4. Here, data taken at both 1.125 and 0.55 MHz display only the collapsed behavior, while the diffusion at 2.24 MHz is compatible with particles whose size corresponds to the pure silica core. At later times (i.e., higher *T*_eff_), all of the datasets deviate from these regimes, suggesting the accumulation of sample damage within a train.

The data show that the combination of high repetition rate and high fluence (F4) completely destroys the shell, while samples probed at lower repetition rates survive these higher dose rates. This effect can be qualitatively understood remembering the fact that the collapse of the shell is a phase transition from a hydrophilic state to a hydrophobic one and that the collapse in itself requires a finite time to happen. At those fluences, each XFEL pulse carries enough energy to heat up the core silica particle and the surrounding volume well above *T*_lcst_ = 312 K. At the same time, it will produce a certain amount of damage, poking holes in the polymer network. If the PNIPAm is still fully hydrated, then the self-diffusivity is high, and it will be easier for damaged sections of the shell to escape and diffuse into the solvent and eventually forming smaller nanogels. Conversely, if the PNIPAm is already in a collapsed state, then it will be more difficult for the damaged parts to escape because of reduced diffusivity within the network and the hydrophobicity. Therefore, looking at the diffusivity of the particles and thus their hydrodynamic radius, it would appear that the collapsed particles maintain a substantially higher damage threshold. When the second pulse reaches a system of already collapsed particles, it will basically heat up the surrounding water similarly to (*25*, *26*). In contrast, if the shells of the particles are not yet collapsed, then it will aggravate the damage, and, after the initial ballistic phase, the majority of the particles will have their shells completely removed. This suggests that the time required for a shell to collapse must be smaller than 0.88 μs (1.125 MHz) but not too different from 0.44 μs (2.24 MHz). Therefore, the diffusion constant is a quantity that depends not only on *T*_eff_ but also on the timescale. A direct consequence of this time-dependency is visible in Fig. 4B, where the 0.55-MHz data reach the “collapsed line” at lower *T*_eff_ than the data taken at 1.125 MHz. For 0.55 MHz, each *T*_eff_ is separated by a larger time interval, allowing a more complete collapse between the pulses. This is in accordance with observations that both the collapsing and swelling times are connected with the diffusion of water in a complex environment such as the polymer network of the PNIPAm shell (*10*, *13*). It is interesting how this damage threshold is associated with a dose-rate rather than a total dose. Previous studies performed at SACLA on similar systems of silica core-PNIPAm shell identified a damage threshold at 4 × 10^2^ kGy (*36*). The damage was observed in changes of the *I*(*q*) showing signs of agglomeration. In the F4 regime, the dose delivered by a single pulse was about 8.8 kGy, and the threshold of (*36*) is reached after 46 pulses. The existence of a total dose threshold is visible also in our dynamical data and can be identified with the violation of the Stokes-Einstein relation in the *D*(*T*_eff_) plots of Fig. 4. In the specific case of 1.125-MHz data, this violation appears at ~57 pulses, thus ~5 × 10^2^ kGy, reported in fig. S4. The violation of the Stokes-Einstein relation in this case is likely due to substantial changes within the scattering volume, e.g., changes in viscosity as a consequence of fragments produced by the damaged PNIPAm or the initial steps of the formation of large aggregates. The mismatch between our threshold and the one reported in (*36*) is due to the fact that the latter experiment was performed at 10 Hz, thus observing timescales much longer than what can be accessed here (few hundreds of microseconds).

**Fig. 5.**
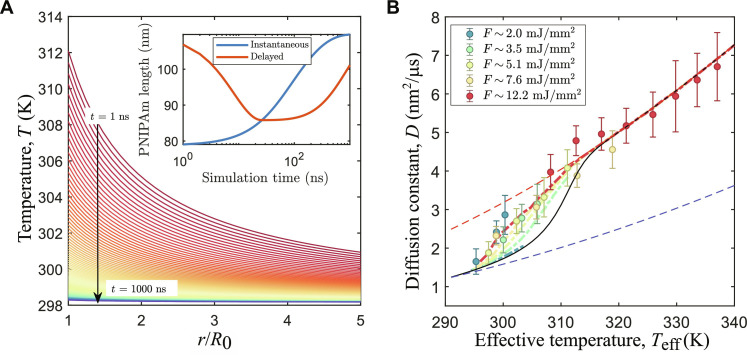
Results from time-resolved model. (**A**) Calculated temperature as a function of *r*/*R*_0_ and *t* after a temperature jump of 14 K. In the inset, the calculated elongations of the PNIPAm chains are reported for a situation in which there is no delay in the response (blue) and a delay of 10 ns and 800 ns in the collapse and swelling, respectively. (**B**) Model results for 1.1-MHz data and thick shell and 8 wt % BIS compared with the experimental data (model: dot-dashed lines; data: circles). The dashed lines are limit values that mark the diffusion of swollen (blue) and collapsed (red) particles. The black line represents the instantaneous response limit.

Next, we take a closer look on the measured *D*(*T*_eff_) for a collapsing PNIPAm shell. Following the reasoning reported in (*12*), we can extend the “time-resolved” model illustrated in (*25*) by adding a temperature- and time-dependent radius. The temperature dependence is obtained by a parametrization of the equilibrium response measured at macroscopic length scales by DLS, while a time dependence is introduced with two exponential relaxations that delays all changes of the PNIPAm length. In Fig. 5A, the calculated local temperature experienced by a nanoparticle is reported. Further details of the temperature model can be found in the Supplementary Materials. In short, assuming a spherical particle, it is possible to analytically describe the temperature as a function of both time and distance from the center of the particle with

$$T\left(t,r\right)=\frac{\Delta T}{r/R_{0}}e^{-\Gamma_{c}t}+T_{0}$$ (2)

where *R*_0_ is the particle’s radius, ΔT is the temperature jump produced by the absorption of the x-rays, *T*_0_ is the temperature before the pulse, and Γ*_c_* is the thermal relaxation rate. In Fig. 5, we use Γ*_c_* = 4.6 MHz and ΔT = 14 K, which are typical values for *F* ≈ 13 mJ/mm^2^. The temperature changes as a function of distance from the particle’s surface, and, eventually, it crosses *T*_lcst_ within the length of the PNIPAm polymer. To account for this situation, the thickness of the PNIPAm polymer [*L_P_*(*t*)] is divided into *N* segments calculated as follows

$$L_{P}\left(t\right)=\sum_{i=1}^{N}\;l_{i}\left[T\left(t,r_{i}\right)\right]$$ (3)

where $r_{i}=\sum_{j=0}^{i}\;l_{j}$ is the sum of all the previous segments starting from the particle’s surface.

In the inset of Fig. 5A, we report the PNIPAm elongation as a function of time. The blue line is the result for a polymer that can follow instantaneously all temperature changes, while the red line is the elongation of a polymer that can collapse and swell exponentially with finite characteristic times τ_collapse_ and τ_swell_. In this example, we set τ_collapse_ = 10 ns and τ_swell_ = 800 ns. The calculated radius is used to compute an instantaneous diffusion constant that is averaged over the time interval between two pulses. In Fig. 5B, the experimental data points for the thick-shell and 8% BIS sample at 1.1 MHz are reported together with the output of the model (dot dashed lines). For the lower fluences of regime F2, the model predicts a behavior similar to the one expected from an instantaneous response of the PNIPAm shell (black continuous line), while the data points show already a partial collapse. At such fluences, the pulses are not strong enough to rise the local temperature above *T*_lcst_, and the particles are affected mostly by the temperature of the surrounding water. Upon increasing the fluence, the predicted diffusivity departs from the “instantanous response” until it reaches a behavior in which the transient temperature gradient surrounding the particles and the internal characteristic times of the polymer shell play a major role, describing a behavior more in agreement to the data. The fact that the data points seem to follow the same master curve, suggests that even weak pulses are able to trigger a collapse of the shell.

**Fig. 6.**
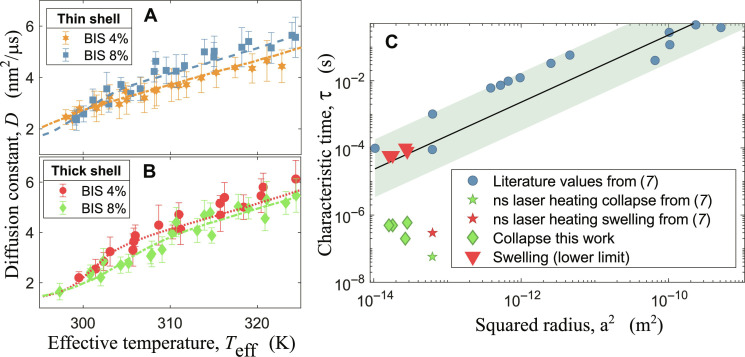
Fitted models and results compared with literature. (**A**) Data for thinner shell and different BIS concentrations, data at 8 wt % BIS (blue squares) are obtained from 1.125-MHz runs, while 4 wt % BIS (orange hexagons) were obtained from 0.55-MHz runs because, for technical reasons, it was not possible to measure F3 data for the latter sample. (**B**) Data for 1.125-MHz runs for thicker shell at different BIS concentrations of 4 wt % (red circles) and 8 wt % (green diamonds). The fits shown here used a fixed τ_swell_ = 80 μs (dashed lines of respective colors). (**C**) Results of the present work, diamonds and triangles, reported together with literature values from (*7*) (stars) and references therein (circles). The line is a linear fit to the literature data and the shaded area is the confidence band. ns, nanosecond.

The model does not provide predictions outside of the time-resolved evolution of the temperature surrounding the particles, and more complete theoretical descriptions are beyond the aim of this work. However, despite its simplicity, it is possible to fit it to the experimental data at F3 fluence regime to estimate the characteristic times τ_collapse_ and τ_swell_ of the polymeric shell. In Fig. 6 (A and B), the data at fluences in the F3 regime are reported together with the corresponding fits of the models for the set of samples. In Fig. 6A, the data for the thinner shell are reported, while Fig. 6B shows the data for the thicker shell. It is possible to track how the collapse is triggered at low *T*_eff_ values in all cases. In those fits, the only free parameters were τ_collapse_ and τ_swell_, while *R*(*T*_eff_) was determined by previous DLS characterization; see Table 1 where the particles’ radii and the results from the fit of τ_collapse_ are reported. It is important to notice that, in the data, the parameters τ_collapse_ and τ_swell_ are correlated. Because the diffusion constant is sampled at a given rate, i.e., the diffusion is only probed at time intervals defined by the EuXFEL repetition rate, every combination of τ_collapse_ and τ_swell_ that gives the same PNIPAm length in the time interval between two pulses will produce the same result. There are, however, two limits of the set of possible solutions for which these two parameters become independent: one for a fast τ_collapse_ (below ∼10 ns) and one for a slow τ_swell_ (above ~80 μs). The former limit corresponds to an almost instantaneous collapse process, while the latter represents a condition in which the system is not able to recover within the time interval between two pulses. Considering that, in the previous section, we concluded that the time required for the shell collapse must be close to 440 ns (2.24-MHz repetition rate), the more likely scenario is the one for slow τ_swell_. Under this assumption, the resulting τ_collapse_ are reported in Fig. 6C. A high concentration of cross-linkers slows down the collapse process, suggesting that a stiffer shell requires more time to reach a collapsed configuration.

**Table 1.** Summary of the parameters of the investigated samples and results from the fitting procedure. The radii are obtained from the DLS characterizations reported in the Supplementary Materials. The values of τ_swell_ are the lower limits for the swelling times.

*R*(*T* = 296 K)	*R*(*T* = 315 K)	BIS %	τ_collapse_	τ_swell_
156 ± 3 nm	83 ± 5 nm	8 wt %	595 ± 50 ns	≥80 μs
157 ± 4 nm	81 ± 8 nm	4 wt %	198 ± 20 ns	≥100 μs
129 ± 3 nm	79 ± 7 nm	8 wt %	496 ± 34 ns	≥60 μs
122 ± 4 nm	86 ± 8 nm	4 wt %	495 ± 22 ns	≥60 μs

According to Tanaka’s model both τ_collapse_ and τ_swell_ should scale with the square of the swollen PNIPAm radius even for core-shell nanoparticles (*11*). In Fig. 6C, we report our results together with the literature values from (*7*) and references therein as well as Tanaka’s model. Roughly speaking, it is possible to group the measurements of the PNIPAm kinetics into two main categories: global heating, where the temperature jump is applied to the water, and local heating, where the temperature jump is applied directly to the particles. It is clear to see that τ_collapse_ deviates from the general trend observed for the global heating experiments (blue circles) and is instead closer to the local heating obtained with ns laser pulses tuned at the plasmon resonance of the core particles. This can be expected because both experiments exploit the local heating of the cores to trigger the phase transition. In contrast to the other studies, we observed a strong difference between swelling and collapse timescales. The lower limits for the swelling times fall close to the points observed for global heating experiments. The x-rays slightly heat up also the water in the scattering volume. Considering that the timescales necessary to dissipate the heat is on the order of hundreds of microseconds [see supporting information of (*26*)], the situation is more similar to the one of global heating experiments. It is worth noticing that, even in the opposite limit of the possible solutions for our fit (τ_collapse_ ∼ 10 ns and τ_swell_ ∼ 1 μs), the results would remain qualitatively the same, with a collapse time close to the local heating experiments and a swelling time close to the global heating ones.

## DISCUSSION

Because of the short wavelengths and the megahertz repetition rates of the EuXFEL, we could access the time and temperature evolution of the diffusion coefficient of silica/core-PNIPAm/shell nanoparticles when the PNIPAm undergoes a solubility phase transition induced by a change in (local) temperature above *T*_lcst_. We observed the presence of two damage thresholds: one related to the accumulated dose that is compatible with the valules observed in previous experiments (*36*) and one that depends both on the dose rate and on the repetition rate, from which we can infer an upper limit on the characteristic collapsing time. It is interesting to point out how the latter damage threshold is observed at much lower total doses. For the sake of discussion, it is interesting to consider also another interpretation of the 2.2-MHz data in fluence regimes F2 and F3. In this interpretation, the particles would not be damaged but transfer in an “over-collapsed” state. In equilibrium conditions, PNIPAm retains a certain amount of water in its volume even above the *T*_lcst_ (*38*). Within this alternative interpretation, the high repetition rates would induce a squeeze out of this remaining water content resulting in particles with an effectively smaller hydrodynamic radius. Consequently, the collapsing time of the PNIPAm shell should be on the order of ∼10 ns, which is still consistent with our model. This “over-collapse” interpretation is, however, highly speculative and would lead to the counterintuitive result that higher dose-rates are, in practice, less harmful to the sample. For this reason, we consider it unlikely, but, nevertheless, we believe that it might be a target of investigation for other experiments.

Adapting a phenomenological model previously developed to this system, we were able to observe that the phase transition happens even at relatively low local temperatures, suggesting an effective lowering of *T*_lcst_, whose origin is still unclear. Given the characteristics of the system, the creation of free charges after the absorption of the x-rays should play a role, while charges in solution lower the *T*_lcst_ (*39*, *40*), charges embedded in the polymer network lead to the opposite effect (*41*, *42*), and the two effects influence each other (*43*). With our model, we were able to fit the data and extract τ_collapse_ and τ_swell_. While τ_collapse_ is consistent with results from laser heating of gold cores of gold-PNIPAm nanoparticles, the swelling times τ_swell_ resemble the ones of global heating experiments. This indicates that the x-ray–induced heating process with successive cooling differs from local and global heating processes studied so far. Moreover, we were able to observe an effect of the concentration of cross-linkers on the kinetic response of the polymer, effectively slowing down the collapse for larger concentrations. It is worth noticing that this kind of information has been obtained from diffusion experiments coupling the possibility to probe nonequilibrium systems with XPCS with the megahertz and nanometer time and length scale reachable at EuXFEL. These findings are important not only for the investigation of the kinetics of the PNIPAm collapse but also for future studies where the collapse and swelling are triggered in more complex environments. More in general, the advancement of these techniques and their succesful application to more and more radiation sensitive systems is a great asset for investigations tackling biologically relevant phenomena such as protein folding kinetics.

## MATERIALS AND METHODS

### XPCS technique

Coherent and partially coherent electromagnetic radiation, once diffused by a random media, produces an interference phenomenon also known as speckle pattern. The speckle pattern encodes the density fluctuations of the material that generated it and can be used to access the intermediate scattering function (*44*). More in detail, in a generic XPCS measurement, one collects the scattered intensity within a given time interval (here determined by the XFEL pulse duration, thus ≈50 fs) at a certain *q*, thus selecting an appropriate region of interest (ROI). One can compute the TTCM [*C_q_*(*n*_1_, *n*_2_)] as follows

$$C_{q}\left(n_{1},n_{2}\right)=\frac{<I_{\text{pix}}\left(n_{1}\right)I_{\text{pix}}\left(n_{2}\right){>}_{\text{pix}}}{<I_{\text{pix}}\left(n_{1}\right){>}_{\text{pix}}<I_{\text{pix}}\left(n_{2}\right){>}_{\text{pix}}}-1$$ (4)

where <…>_pix_ denotes the average over the pixels within the same *q*-ROI, *n*_1,2_ indicates the number of frames collected since the beginning of the measurement, and *I*_pix_(*n*) is the intensity recorded at pixel pix at frame number *n*. Typically, one finds the TTCM expressed as a function of time that is obtained straightforwardly via *t*_1,2_ = δ*tn*_1,2_, where δ*t* is the time interval between two frames. For stationary systems, the dynamics depends only on lag time *t* = ∣*t*_2_ − *t*_1_∣, and it is possible to average *C_q_*(*t*_1_, *t*_2_) along its diagonals in order to obtain the more common autocorrelation function *g*_2_(*t*) − 1. It can be demonstrated that *g*_2_(*t*) − 1 is proportional to the square modulus of the intermediate scattering function, with a proportionality constant given solely by experimental parameters (*34*). In general one can describe *g*_2_(*t*) − 1 with a KWW function (or stretched exponential) *g*_2_(*t*) − 1 = β*_q_* ⋅ exp [−(*t*/τ)^γ^]. The simple diffusion is a particular case of this KWW function where γ = 1 and 1/τ = *q*^2^*D*. For nonstationary dynamics, the TTCM ceases to depend solely on *t* and will become a function of another time, often indicated as the “age” *t_w_*. There are several ways to map the *C_q_*(*t*_1_, *t*_2_) into the *C_q_*(*t_w_*, *t*); see, for example, (*30*); here, the convention *t_w_* = *t*_1_ and *t* = *t*_2_ − *t*_1_ is followed. For nonstationary dynamics with age-dependent parameters, we can then write (*45*, *46*)

$$C_{q}\left(t_{w},t\right)=\beta_{q}\text{exp}\left\{-[{t/\tau \left(q,t_{w}\right)]}^{\gamma }\right\}$$ (5)

To take in account the change in shape of the relaxations, the average relaxation time is considered, obtaining a set of <τ> for each *t_w_* and *q*. Then, each set of <τ> is fitted by 1/[*D*(*t_w_*)*q*^2^], and, to discriminate between the ballistic-like and diffusive dynamics, the fits with a coefficient of determination (*R*^2^) lower than 0.85 were rejected. Then, the diffusion constant is calculated again for each accepted <τ(*q*, *t_w_*)>, and the SD is used to estimate the uncertainty over *D*(*t_w_*).

### Synthesis details

Synthesis of silica nanoparticles: 352 ml of ethanol, 3.4 ml of ammonium hydroxide (28 to 30%), and 54 ml of ultrapure water were vigorously stirred in a 1-liter Erlenmeyer flask. Afterward, 29.0018 g of tetraethyl orthosilicate and 60 ml of ethanol were added quickly and the reaction was let to proceed for ~24 hours. In the next step, coating with 3-trimethoxysilyl propyl methacrylate was undertaken. For that, 8 ml of 3-trimethoxysilyl propyl methacrylate were dissolved in 10 ml of ethanol and added dropwise to the reaction flask. Reaction was let to proceed for another 24 hours. After that, the suspension was concentrated using a rotational evaporator and then dialized against ethanol for 1 week. The whole procedure was carried out at room temperature. A portion of these silica nanoparticles in ethanol was measured in a separate SAXS experiment performed at a syncrotron beamline (P10 at PETRA III). From that characterization, it was possible to determine that the core particles are spheres with average radius *R*_0_ = 43.3 ± 0.1 nm and size distribution ΔR/*R*_0_ = 0.10 ± 0.01.

For the shell synthesis: 500 ml of water was heated up to 60°C in an oil bath. Then, under nitrogen flow, a mixture of 0.126 g of sodium dodecyl sulfate and 0.139 g of sodium sulfite together with 15 ml of the silica nanoparticles dispersed in ethanol was injected. Everything was stirred at 60°CC under nitrogen for 1 hour. After that, 0.135 g of potassium peroxodisulfate and 4 mg of ammonium iron(II) sulfate hexahydrate dissolved in 3 to 5 ml of water were added. Immediately afterward, 1 g (1.5 g) of *N*-isopropylamide, for the thin and thick shells, respectively, and the corresponding quantities of *N*,*N*′-methylenbisacrylamide to reach the BIS concentrations of 4 and 8 wt % were dissolved in 20 ml of ethanol and added dropwise, and the overall time for the addition was ~10 min. The reaction was left to proceed for 24 hours, and, after that, the suspension was concentrated and dyalized against water for 1 week. The dispersions used for the results reported here had mass concentrations between 3 wt % (thin shell, 4 wt % BIS) and 4 wt % (thick shell, 8 wt % BIS). The corresponding volume fractions in the swollen state were ~12 and ~25% for the thin and thick shell, respectively, while, in the collapsed state, the volume fractions were ~2.8 and ~3.5%. In all cases, the concentration was low enough to have freely diffusing particles with no short-range order, i.e., *S*(*Q*) ≈ 1, as can be inferred from (*24*).

Last, the colloidal dispersions were filled into thin-walled quartz capillaries (Hilgenberg GmbH, wall thickness 10 µm) with an outer diameter of 1.5 mm that were sealed and placed in a specifically designed sample holder. The experiment was performed in air at the MID instrument. A detailed description of the instrument can be found in (*47*).

### Setup

The experiment has been carried out in two rounds; in both rounds, the photon energy was set to 9 keV. The intensity of the x-ray pulses was measured with a gas monitor placed upstream the sample position. The beam-size at the sample position was about 8 and 10 μm in diameter in the first and second round, respectively, obtained by focusing the beam by compound refractive lenses. The x-ray fluence was controlled with stacks of chemical vapor deposition (CVD) diamond plates that could be inserted to absorb the beam. For the data reported here, the total thicknesses ranged from 2.5 to 7.05 mm. Taking the beamline and air transmission into account, the intensities on sample were reduced by a factor ranging from 1.2 × 10^−4^ to 1.5 × 10^−3^ with respect to the one measured with the gas monitor. The scattered intensity was recorded with an adaptive gain integrating pixel detector mounted downstream the sample. Distance and relative positions of the four detector quadrants were determined with the aid of a known reference sample measurement measured also at a well-characterized synchrotron beamline (*37*). A sample-detector distance of 7.381 m was determined this way. The samples were placed in sealed quartz capillaries and measured in air at room temperature.
