# Supplementary material for: Real-time swelling-collapse kinetics of nanogels driven by XFEL pulses
Source: Sci Adv. 2024 Apr 19;10(16):eadm7876. doi: 10.1126/sciadv.adm7876 (PMC11029799; doi:10.1126/sciadv.adm7876)
Supplement: Supplementary file 1 — Supplementary Text Figs. S1 to S11 Table S1 [file sciadv.adm7876_sm.pdf]

Science Advances
AAAS

Supplementary Materials for

Real-time swelling-collapse kinetics of nanogels driven by XFEL pulses

Francesco Dallari *et al.*

Corresponding author: Francesco Dallari, francesco.dallari@unipd.it;
Felix Lehmkühler, felix.lehmkuehler@desy.de

Sci. Adv. **10**, eadm7876 (2024)
DOI: 10.1126/sciadv.adm7876

This PDF file includes:

Supplementary Text
Figs. S1 to S11
Table S1

Calibration with pure silica particles

The calibration was performed with highly monodisperse silica particles previously characterized in other synchrotron experiments. The comparison of the I(q) measured at synchrotron light-sources, reported in ref (33), allowed us to obtain a precise measurement of the sample-detector distance, while investigating the heating effect at different fluences and repetition rates, see figure S1, allowed us to have a reliable estimation of the actual fluence impinging onto the sample.

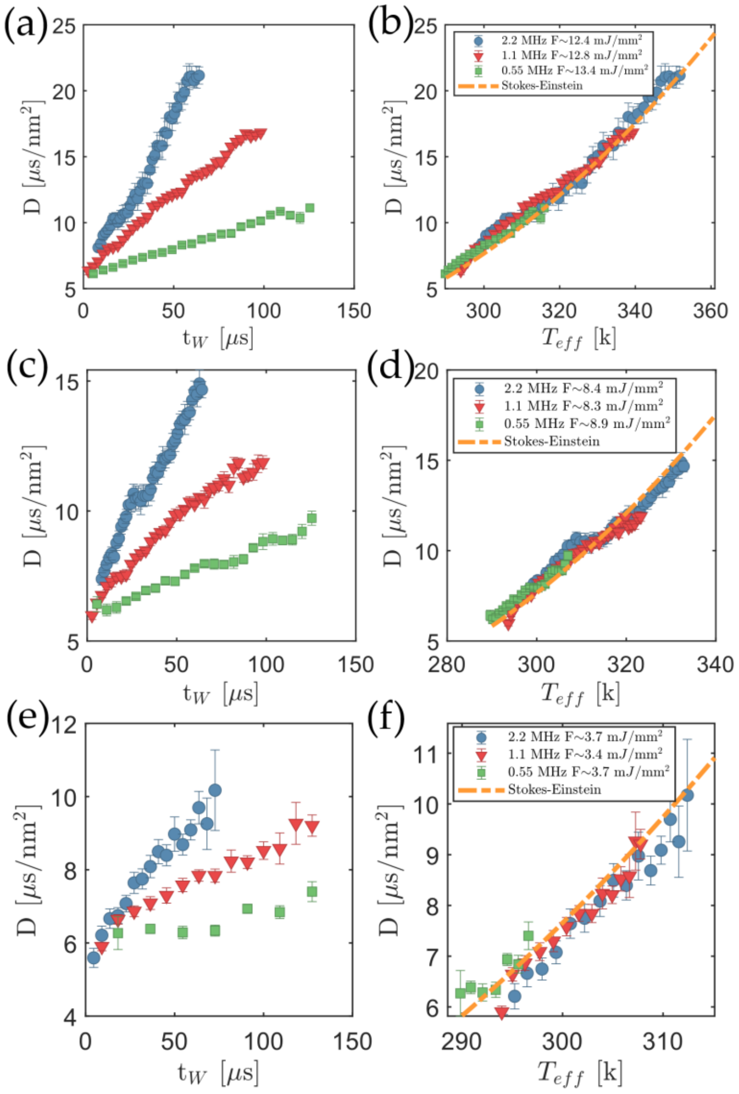

Fig. S1.

Calibration of the transmittance of the MID beamlin. Once the correct value is obtained then the prediction of the “time resolved model” of ref (25,26) allows us to calculate an effective temperature that satisfies the Stokes-Einstein relation for all combinations of fluence or repetition rate. Left hand side (a, c, e): Diffusion constants as a function of experimental time for high (a), medium (c) and low (e) fluences. Right hand side (b,d,f) same diffusion constants reported as a function of their respective calculated effective temperatures. The orange dashed lines are the expected Stokes-Einstein relations for those particles.

I(q) from silica-PNIPAm particles

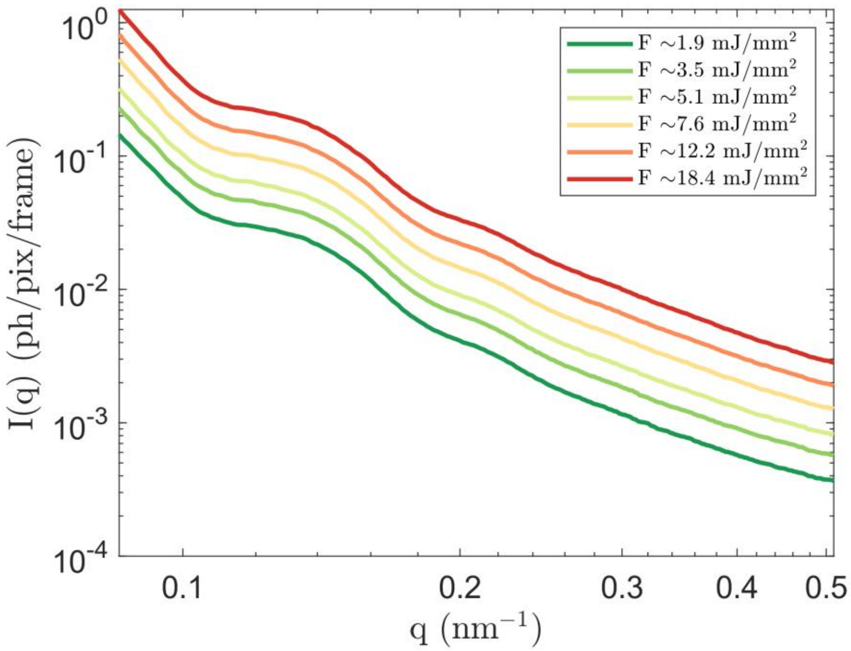

Fig. S2.

Example of the intensity scattered from samples with BIS 8 wt%. The intensities are obtained averaging over 200 frames in a measurement. It is possible to see that the main features of the I(q) do not change with fluence. The only visible effect is a small change in the slope at larger q (range ~ 0.19 nm^{-1} - 0.46 nm^{-1}), but the low count-rate in that range makes it difficult to extract quantitative information.

Effects of radiation on I(q)

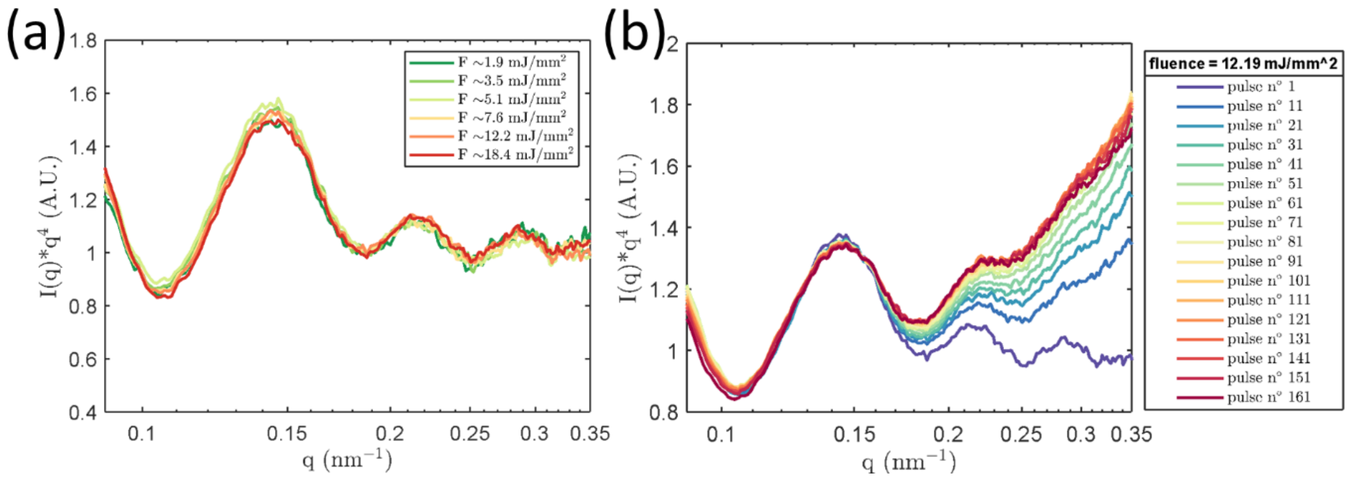

Fig. S3.

Rescaled scattered intensity multiplied by q^4 to highlight the details at larges q values. (a) curves obtained considering the first 10 pulses within a train for different fluences; despite changing the pulse intensity, the nanoparticles maintain the same shape. (b) dependence on number of pulses in the normalized $I(q)*q^4$ at moderate fluence; it is possible to see that the Porod behavior is progressively lost in accordance with ref (37) of the main text where the building up the damage produced a transition from $I(q) \propto q^{-4}$ to $I(q) \propto q^{-3}$.

Diffusion constants as a function of number of pulses

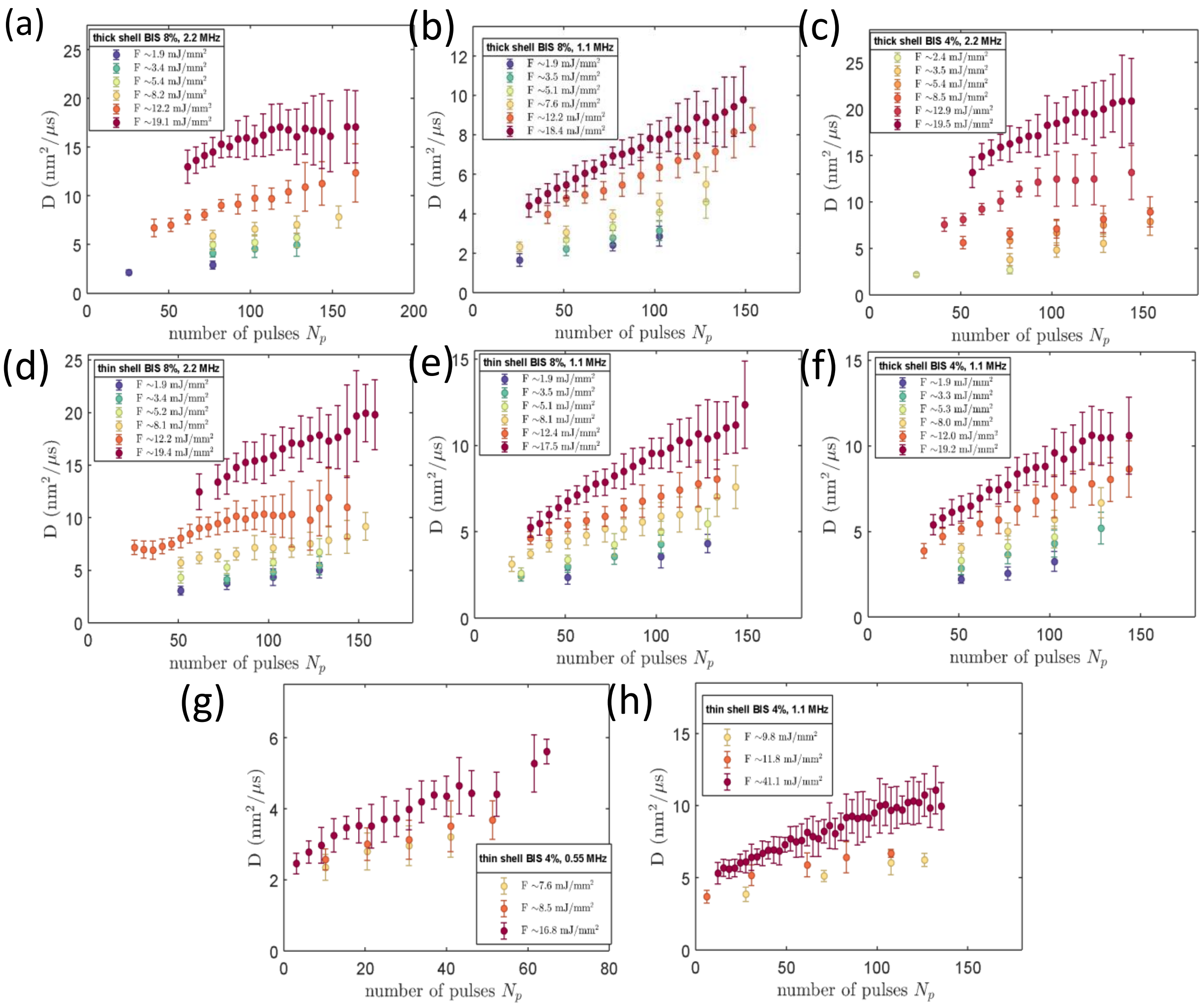

Fig. S4.

Diffusion constants as a function of N_P for the datasets used in the manuscripts. Measurements at 1.1 MHz (b,e,f,h) and 2.2 MHz (a,c,d) for the samples with BIS concentration of 4wt\% (c,f,g,h) and 8 wt% (a,b,d,e).

Damage threshold of the cumulative dose

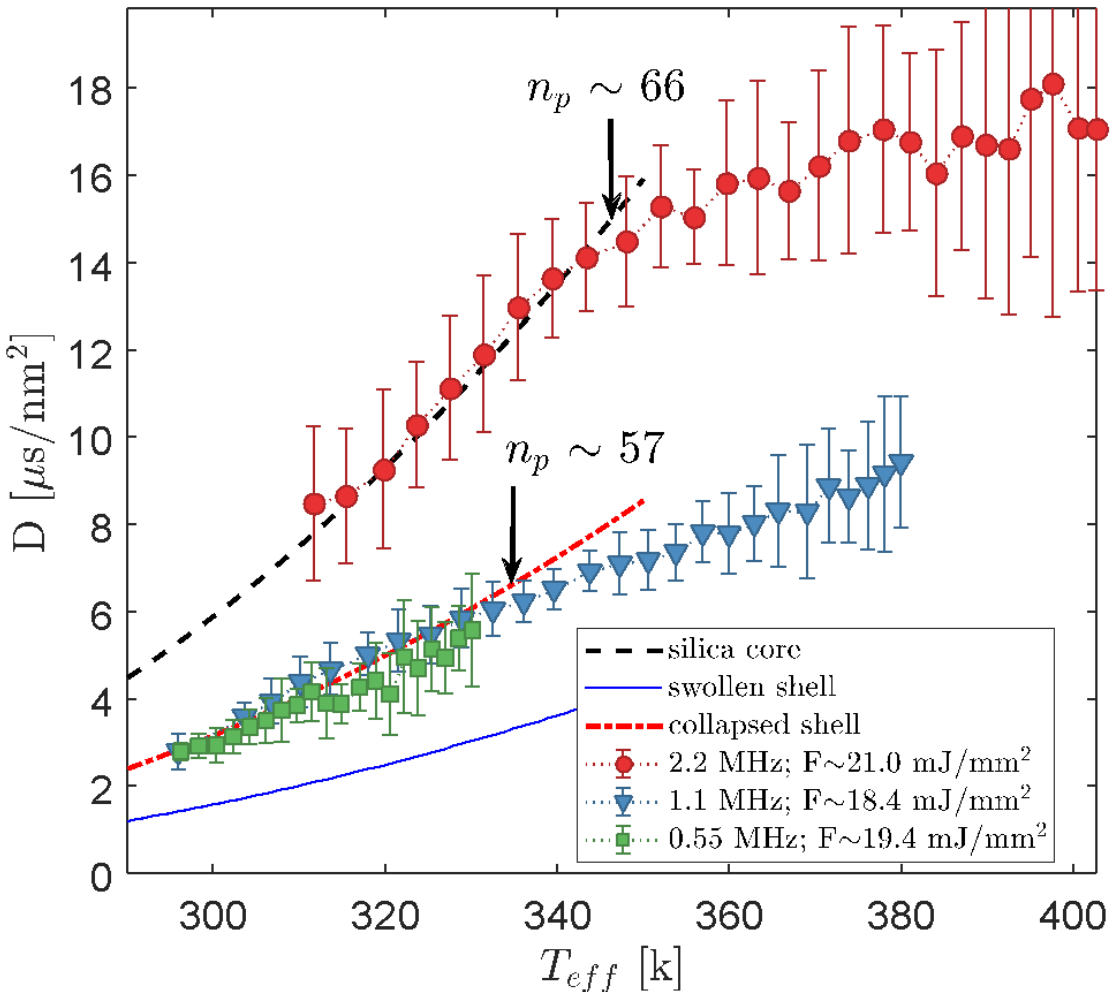

Fig. S5.

Cumulative damage threshold for fluence F4. Both the 1.1 MHz and 2.2 MHz follow the expected Stokes-Einstein relation until 57 and 66 pulses respectively resulting in doses of ~$5*10^5$ Gy - ~$6*10^5$ Gy.

Time-temperature dependency of particle's hydrodynamic radius

As reported in the main text, we can extend the "time-resolved" model of ref (25,26) adding an additional temperature- and time- dependent radius. Assuming a spherical particle, it is possible to analytically describe the temperature as a function of both time and distance from the center of the particle with:

$$T(r,t) = \frac{\Delta T}{r/R_0} e^{-\Gamma_0 t} + T_0$$

where R_0 is the particle's radius, ΔT is the temperature jump produced by the absorption of the X-rays, T_0 is the temperature before the pulse and Γ_0 is the thermal relaxation rate. The temperature changes as a function of distance from the particle's surface and eventually it crosses the LCST within the length of the PNIPAm polymer. Therefore, different sections of the PNIPAm will be either above or below the T_{lcst} and will collapse or swell accordingly.

It is possible to parametrize the temperature-dependence of the PNIAPm measured with the DLS data with a sigmoidal function such as:

$$R(T) = R_{sw} + \frac{R_{col} - R_{Sw}}{(1 + e^{-G(T - T_{lcst})})^{0.25}}$$

where R_{sw} and R_{col} are the swollen and collapsed radii respectively whose value is reported in table 1 of the main text, while in this context G is just a parameter.

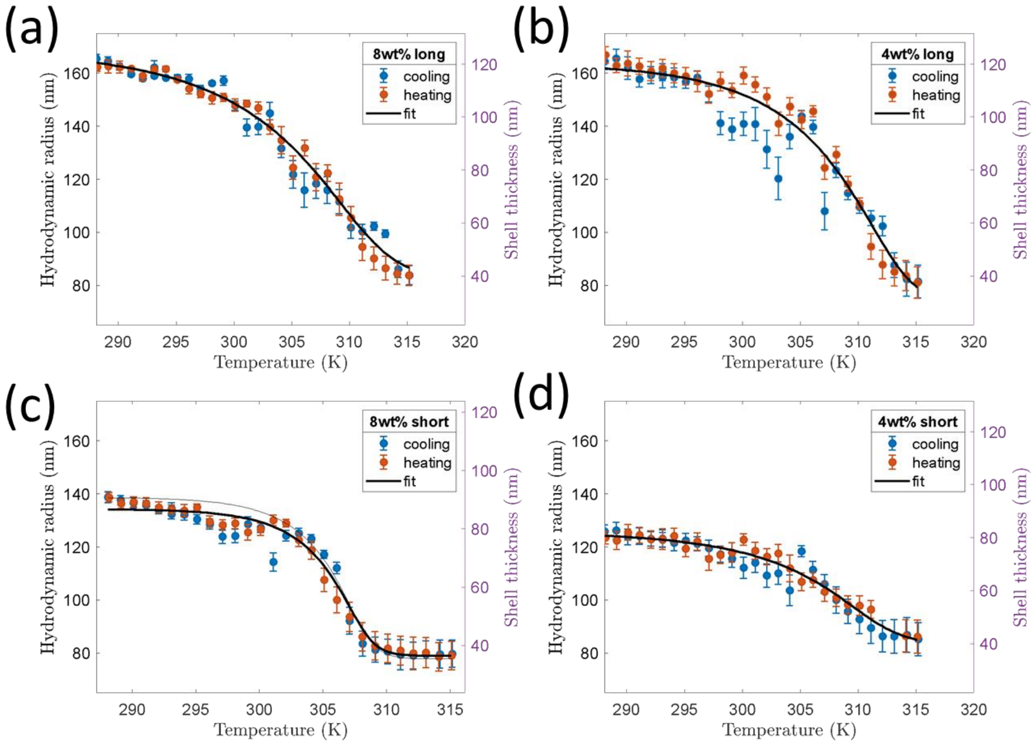

Fig. S6.

DLS characterization. DLS measurements have been carried out on dispersions at the same concentration as the one investigated in the main text. Both cooling and heating data points were fitted with the same phenomenological sigmoidal function. $T_{lcst} \approx 312$ for all samples and $G \approx 0.6$ for all samples except the 8wt% thin shell, where it is ~ 1.1 .

A single segment of PNIPAm at initial length l_0 that finds itself at a certain distance r will be exposed to a temperature T. If it is be at equilibrium its temperature dependence is given by :

$$l^{eq}(T) = l_0 + \frac{l_{coll} - l_0}{(1 + e^{-G(T-T_{lcst})})^{0.25}}$$

However, here the time plays an important role, and the segment length between two pulses will be described by:

$$l(T,t) = l^{eq}(T) - \left(l^{eq}(T) - l(T,t-\delta t)e^{-t/\tau_s} \right) \quad \text{if} \quad l(T,t-\delta t) > l^{eq}(T)$$

or

$$l(T,t) = l^{eq}(T) - \left(l^{eq}(T) - l(T,t-\delta t)e^{-t/\tau_c} \right) \quad \text{if} \quad l(T,t-\delta t) < l^{eq}(T)$$

where δt is the time step of the model (here chosen to be 1 ns), τ_c is the collapsing characteristic time and τ_s the swelling characteristic time.

The total length of a PNIPAm polymer ($L_P(t)$) composed by N segments in a time and space dependent temperature gradient will be given by the sum of all those segments which can be calculated recursively:

$$L_P(t) = \sum_i^N l_i(T(t, r_i), t)$$

Where $r_i = \sum_j^i l_j(T(t, r_j), t)$ is the sum of all the previous segments starting from the particle's surface.

Sample	R_{sw}	R_{col}	G	T_{lcst}
Thick shell BIS 8wt%	168 ± 5	82 ± 8	0.46 ± 0.02	312 ± 1
Thick shell BIS 4wt%	163 ± 5	72 ± 15	0.6 ± 0.2	313 ± 2
Thin shell BIS 8wt%	134 ± 2	78 ± 2	1.1 ± 0.3	308 ± 2
Thin shell BIS 4wt%	126 ± 5	83 ± 5	0.5± 0.1	312 ± 2

Table. S1.

Fit parameters for the DLS characterization.

Correlation between $\tau_{collapse}$ and $\tau_{swelling}$ in the fit results

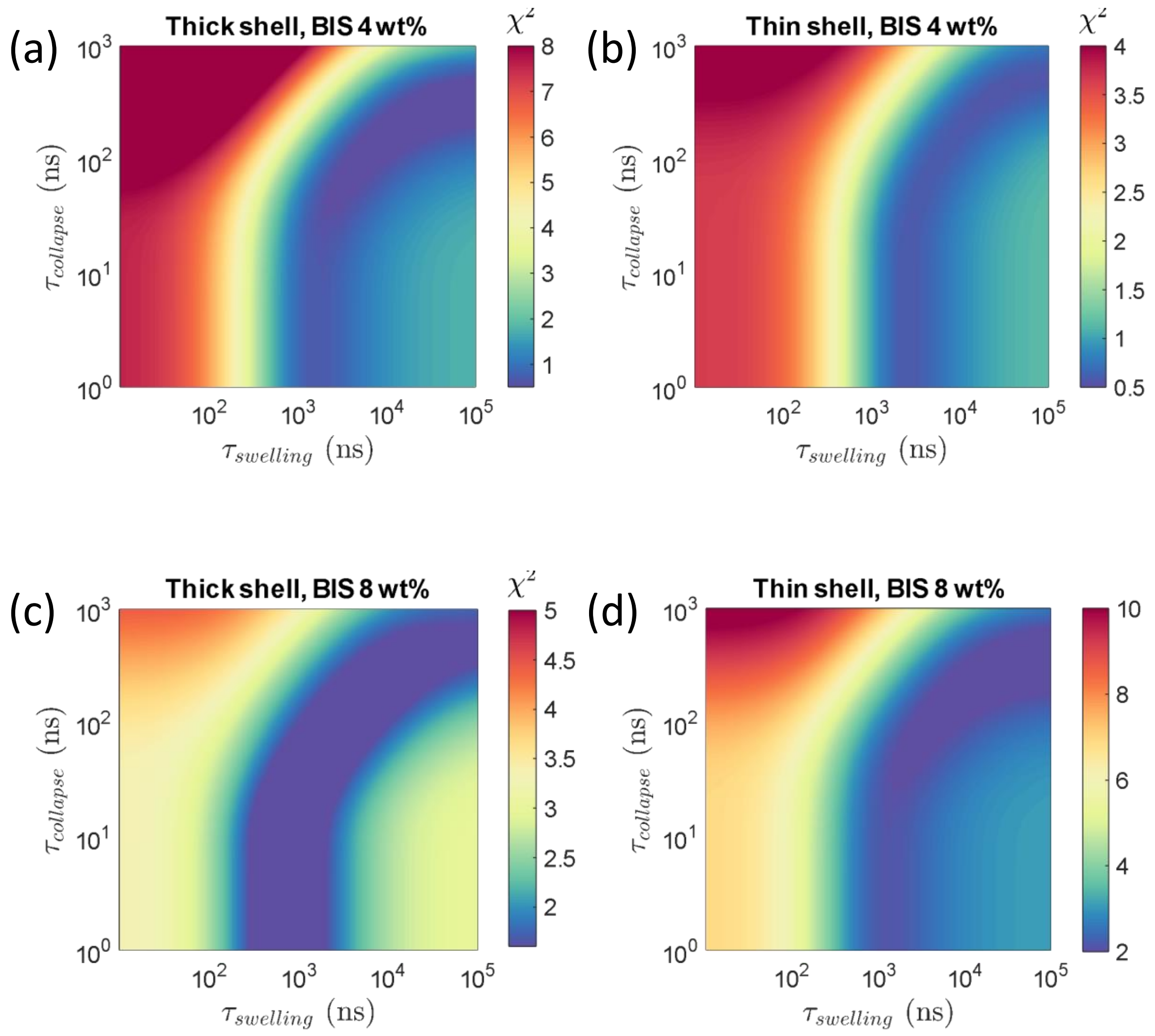

Fig. S7.

χ^2 values for the fit of $\tau_{collapse}$ and $\tau_{swelling}$. It is possible to see how the minimum does not correspond to a unique value of $\tau_{collapse}$ and $\tau_{swelling}$ but rather corresponds to an area that connects the limit for very fast $\tau_{collapse}$ and very slow $\tau_{swelling}$.

Two-Times correlation matrices for a low and high fluence runs

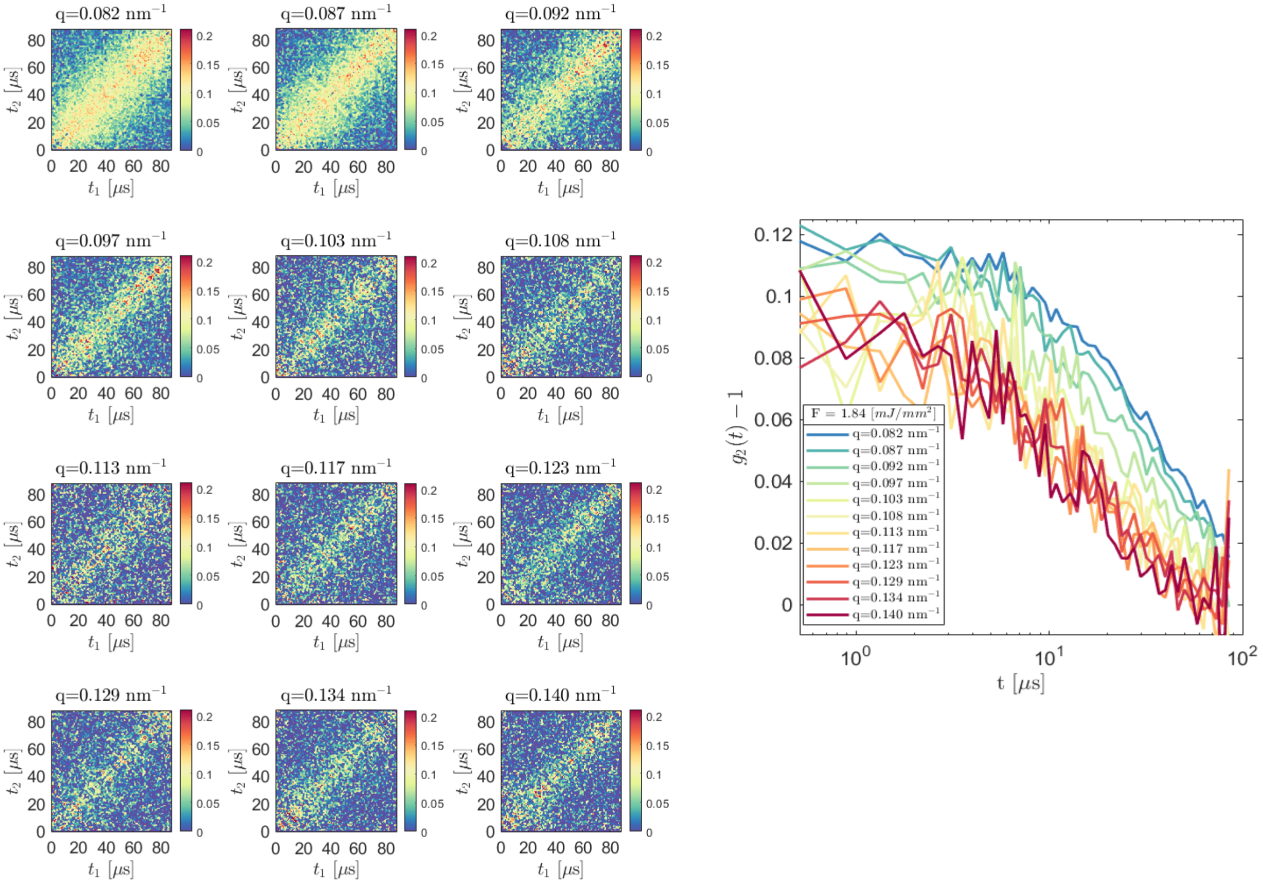

Fig. S8.

Set of two-times correlation matrices and correlation functions for a F1 run on the thick shell BIS 8wt%. On the left, a correlation matrix for each different q, on the right the respective autocorrelation functions.

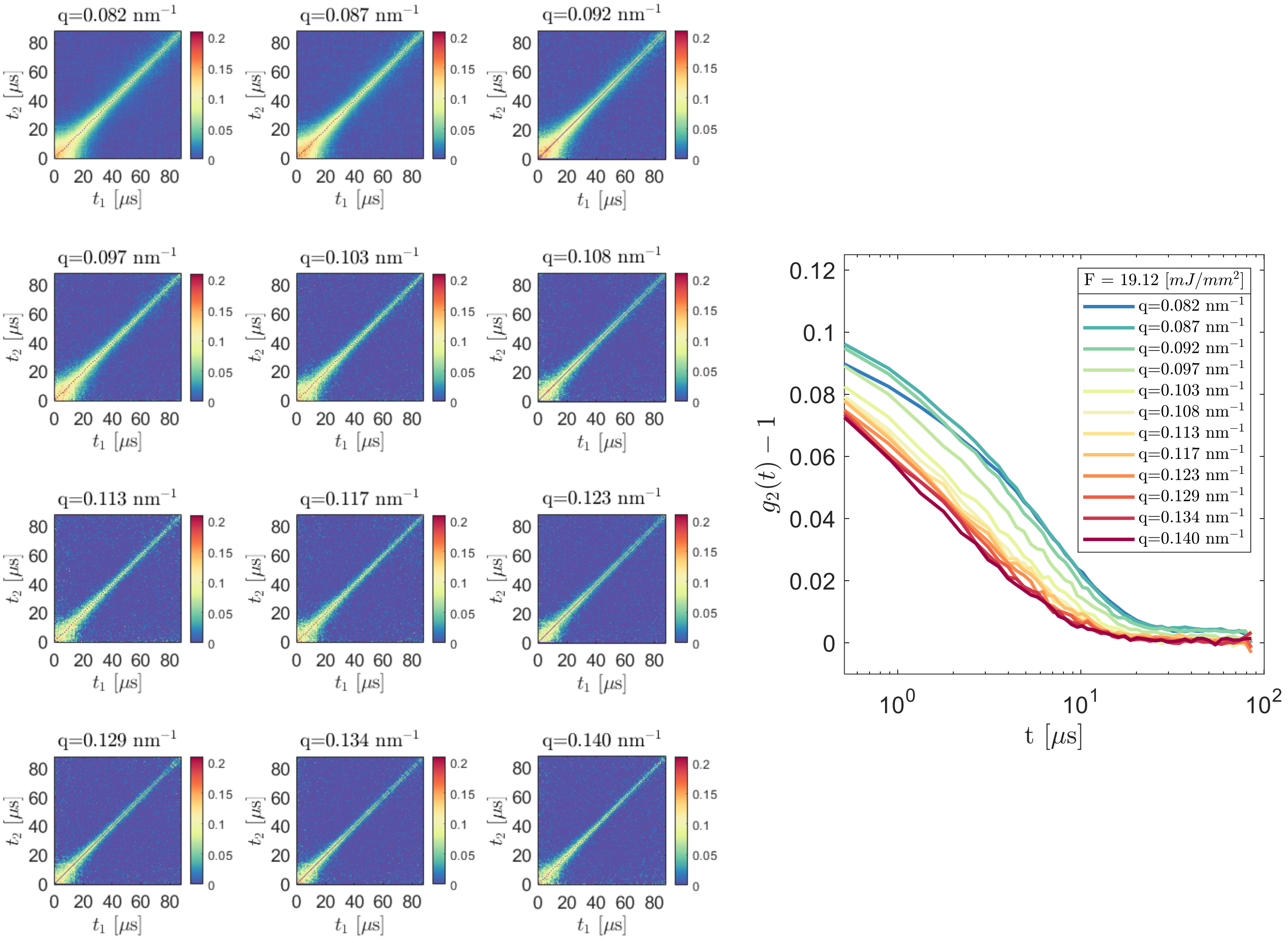

Fig. S9.

Set of two-times correlation matrices and correlation functions for a F3 run on the thick shell BIS 8wt%. On the left, a correlation matrix for each different q, on the right the respective autocorrelation functions.

Fit of the form factor of pure core particles from synchrotron data

An aliquot of the silica nanoparticles dispersed in ethanol was preserved and measured at a later moment at the synchrotron beamline P10 at PETRA III. The form factor was collected with a photon energy of 8.1 keV and a sample to detector distance of 5050 mm. The curve has been fitted assuming a Schulz-Flory distribution of the particle radii. The average radius is R_0 = (43.3 ± 0.1) nm and the resulting dispersity is $\Delta R/R_0$ = 0.10 ± 0.01.

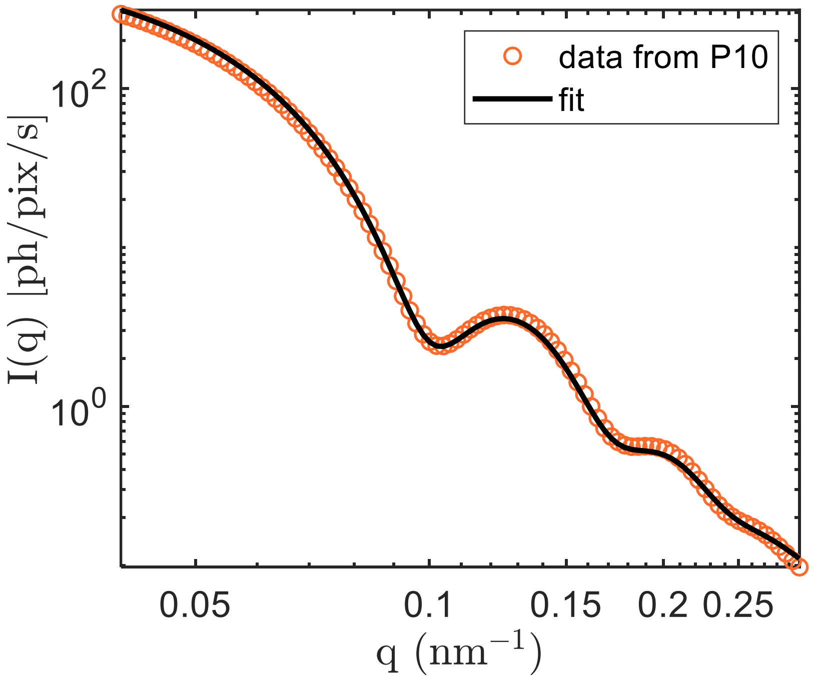

Fig. S10.

Form factor of the core particles. Red circles: Measured scattered intensity from silica particles dispersed in ethanol used to synthetize the core-shell system investigated in the main text. Black solid line: least square fit of a spherical form factor assuming a Shulz-Flory distribution of the particle radii.

I(q) from synchrotron measurements

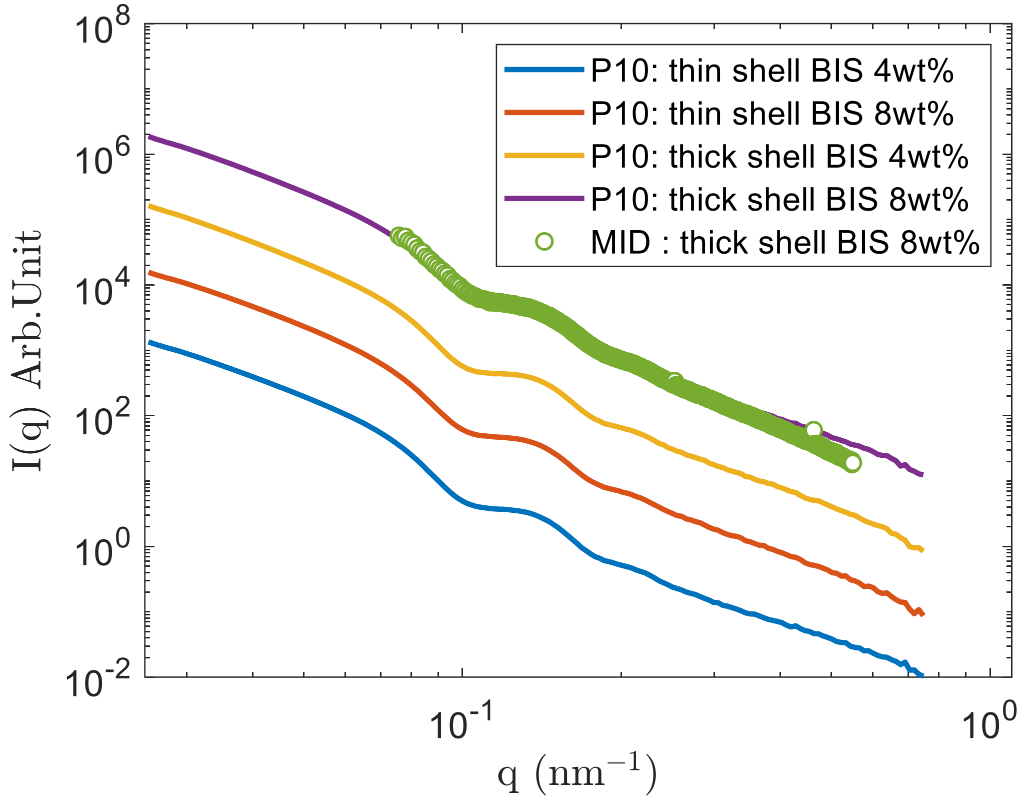

Fig. S11.

Scattered intensity on an extend q-range from synchrotron measurements. Continuous lines I(q) obtained from SAXS experiments on the beamline P10 at PETRA III, circles: data from the experiment performed at XFEL. The PNIPAm shell has a scattering length density only slightly larger than the one from the solvent with the effect that even in a larger q-range the signal is dominated by the scattering from the core particles and no appreciable differences can be noticed for different shell thicknesses and compositions.
